# Genetic background modifies neurodegeneration and neuroinflammation driven by misfolded human tau protein in rat model of tauopathy: implication for immunomodulatory approach to Alzheimer's disease

**DOI:** 10.1186/1742-2094-7-64

**Published:** 2010-10-12

**Authors:** Zuzana Stozicka, Norbert Zilka, Petr Novak, Branislav Kovacech, Ondrej Bugos, Michal Novak

**Affiliations:** 1 Institute of Neuroimmunology, Slovak Academy of Sciences, AD Centre, Dubravska cesta 9, 845 10 Bratislava, Slovak Republic; 2 Axon-Neuroscience GmbH, Rennweg 95b, A -1030 Vienna, Austria

## Abstract

**Background:**

Numerous epidemiological studies demonstrate that genetic background modifies the onset and the progression of Alzheimer's disease and related neurodegenerative disorders. The efficacious influence of genetic background on the disease pathway of amyloid beta has been meticulously described in rodent models. Since the impact of genetic modifiers on the neurodegenerative and neuroinflammatory cascade induced by misfolded tau protein is yet to be elucidated, we have addressed the issue by using transgenic lines expressing the same human truncated tau protein in either spontaneously hypertensive rat (SHR) or Wistar-Kyoto (WKY) genetic background.

**Methods:**

Brains of WKY and SHR transgenic rats in the terminal stage of phenotype and their age-matched non-transgenic littermates were examined by means of immunohistochemistry and unbiased stereology. Basic measures of tau-induced neurodegeneration (load of neurofibrillary tangles) and neuroinflammation (number of Iba1-positive microglia, their activated morphology, and numbers of microglia immunoreactive for MHCII and astrocytes immunoreactive for GFAP) were quantified with an optical fractionator in brain areas affected by neurofibrillary pathology (pons, medulla oblongata). The stereological data were evaluated using two-way ANOVA and Student's t-test.

**Results:**

Tau neurodegeneration (neurofibrillary tangles (NFTs), axonopathy) and neuroinflammation (microgliosis, astrocytosis) appeared in both WKY and SHR transgenic rats. Although identical levels of transgene expression in both lines were present, terminally-staged WKY transgenic rats displayed significantly lower final NFT loads than their SHR transgenic counterparts. Interestingly, microglial responses showed a striking difference between transgenic lines. Only 1.6% of microglia in SHR transgenic rats expressed MHCII in spite of having a robust phagocytic phenotype, whereas in WKY transgenic rats, 23.2% of microglia expressed MHCII despite displaying a considerably lower extent of transformation into phagocytic phenotype.

**Conclusions:**

These results show that the immune response represents a pivotal and genetically variable modifying factor that is able to influence vulnerability to neurodegeneration. Therefore, targeted immunomodulation could represent a prospective therapeutic approach to Alzheimer's disease.

## Background

Alzheimer's disease (AD) is characterized by progressive neurodegeneration of the central nervous system. While the precise aetiology of this disease still remains unknown, it is believed that the intracellular accumulation of hyperphosphorylated tau, which forms neurofibrillary tangles, and the deposition of extracellular filaments, comprised of an insoluble form of the β-amyloid protein (Aβ), induces neurodegeneration. From a molecular perspective, AD is a multifactorial disorder with associations of genetic and environmental factors [1,2]. The onset and progression of AD may be influenced by several risk factors such as hypertension, metabolic disorders like diabetes or hypercholesterolemia, and inflammatory status [3-5].

Numerous studies on several amyloid mouse models of Alzheimer's disease have demonstrated the importance of genetic background for the expression of the transgenic phenotype. Significant influences on survival, behaviour, amyloid levels and plaque burden in brain have been observed [6-10]. Several modifier loci related to these differences have been identified [11-13]. Moreover, genetic background-dependent immunological parameters also modify the effects of amyloid immunization [14,15]. In contrast to investigated amyloid AD models, the role of genetic background in tau-induced neurodegeneration has stayed largely unexplored.

In order to identify the impact of genetic background on the tau neurodegenerative cascade, we generated a transgenic rat model expressing human truncated non-mutated tau protein in the spontaneously hypertensive rat (SHR) and Wistar-Kyoto (WKY) background. The SHR strain was chosen because of its propensity for developing several AD risk factors such as chronic hypertension [16], metabolic syndrome with insulin resistance [17] and immune alterations [18]. Previously, we showed that transgenic SHR rats displayed pathological changes such as the AD-characteristic tau cascade consisting of tau hyperphosphorylation, formation of sarcosyl-insoluble tau complexes and neurofibrillary tangles (NFTs) [19] accompanied with neuroinflammation [20] that resulted in progressive neurobehavioral impairment [21,22]. The transgenic phenotype escalates in the terminal stage with pronounced neurological impairment, hunched posture, muscular weakness, bradykinesia and paraparesis [21]. For this comparative study, we used the normotensive Wistar-Kyoto strain from which the SHR strain was derived. To maintain the same integration site and number of copies of the transgene, transgenic SHR rats were back-crossed to the WKY background.

In this study, we show that misfolded tau proteins induce neurofibrillary degeneration regardless of background strain. On the other hand, genetic background had a significant impact on the pattern of inflammatory response and the final load of neurofibrillary tangles.

## Methods

### Animals

SHR 72 transgenic rats (referred to as SHR TG) were generated to over-express truncated tau protein under the control of the mouse Thy-1 promoter, as described previously [19]. The WKY 72 transgenic line (WKY TG) was developed by back-crossing the SHR 72 line to the WKY genetic background. This study was performed on the F5 generation.

Rats were born and bred in our animal facility, and housed in standard laboratory conditions in plastic cages (555 × 345 × 195 mm, 5 rats per cage) in a temperature and humidity-controlled environment with a 12/12 hour light/dark cycle and with food and water available ad libitum. Efforts were made to minimize the number of animals utilized. All experiments were performed in accordance to the Slovak and European Community Guidelines, with the approval of the Institute's Ethical Committee and State Veterinary and Food Administration of the Slovak Republic.

### Blood pressure

Systolic blood pressure was measured using indirect tail-cuff method (LE 5002 Storage pressure meter, BIOSEB) on the tail arteries of 5 animals of each transgenic and non-transgenic group at 6 months of age.

### Quantitative Western blot analysis

Four-month-old animals, SHR TG males (N = 4) and WKY TG males (N = 4) were used for quantitative western blot analysis. Frozen brain stems of the studied rats were homogenized in 10 volumes of ice-cold extraction buffer [in mm: Tris, 20, pH 7.4; NaCl, 150; ethylenediaminetetraacetic acid (EDTA), 1; dithiothreitol, 1; 0.5% Triton X-100; Na_3_VO_4_, 1; NaF, 20; supplemented with protease inhibitors Complete^® ^without EDTA (Roche Diagnostics GmbH, Mannheim, Germany) using an OMNI TH tissue homogenizer (OMNI International, Marietta, GA, USA)]. After incubation on ice for 5 min the homogenates were cleared by centrifugation at 20 000 g for 20 min at 4°C. The supernatants were collected and the total protein concentration was determined using the BioRad protein Assay (BioRad Laboratories GmbH, Munich, Germany). The supernatants were then mixed with equal volume of 2 × sodium dodecyl sulphate (SDS) sample loading buffer [23] and heated at 95°C for 5 min. Ten micrograms of total protein was separated by electrophoresis in 12% SDS-polyacrylamide gels and then transferred to nitrocellulose membrane in 10 mm N-cyclohexyl-3-aminopropanesulphonic acid (CAPS; pH 12). After the transfer, the membranes were stained with Ponceau S to verify the uniform transfer of the proteins and then incubated with cell culture supernatant (1:5) of Hybridoma-producing pan-tau monoclonal antibody DC25 recognizing residues 347-354 (Axon Neuroscience, Vienna, Austria), followed by polyclonal goat anti-mouse IgG, horseradish peroxidase-conjugated (1:10 000; Dako, Glostrup, Denmark). The blots were developed using Super- Signal West Pico Chemiluminescent Substrate (Pierce Biotechnology, Rockford, IL, USA) and detected with LAS3000 imaging system (FUJI Photo Film Co., Ltd, Tokyo, Japan). The band intensities were quantified using aida (Advanced Image Data Analyser software; Raytest, Straubenhardt, Germany).

### Gallyas silver staining

To demonstrate the mature neurofibrillary pathology in neurons, Gallyas silver iodide staining was performed [24]. Sections were examined with an Olympus BX51 microscope and microphotographs were created with Olympus camera DP-50.

### Frozen sections immunohistochemistry with postfixation

This method was used for the identification of the cell surface markers sensitive to paraformaldehyde fixation. Rats (5 animals per group, transgenic rats in the terminal stage of phenotype with age-matched non-transgenic controls) were deeply anaesthetised with ketamine-xylasine and perfused intracardially with phosphate-buffered saline (PBS) for 2 min using a peristaltic pump. After perfusion, the brainstem was removed and embedded without fixation in cryostat embedding medium (Leica) in aluminium foil vessel, and frozen above the surface of liquid nitrogen until the medium solidified. The sample was left for 1 hour on dry ice, and then stored at -70°C until used. 10-μm-thick sections were cut on a cryomicrotome (Leica CM 1850), affixed onto poly-L-lysine-coated slides, and left to dry at room temperature for 1 hour. Sections were fixed for 10 min in a 80% acetone:20% ethanol solution. Immunostaining was performed using the standard avidin-biotin-peroxidase method (Vectastain ABC kit, Vector Laboratories, USA). After 20 min in 1% H_2_O_2 _and 1 hour blocking in 5% bovine serum albumin (Sigma), sections were incubated with primary antibodies OX-42 (CD11b/CD18) and W3/25 (anti-CD4) (Serotec, UK) overnight at 4°C. After washing, the sections were incubated 1 hour in biotinylated secondary antibody (Vectastain ABC kit). The reaction product was visualized using avidin-biotin and Vector VIP as chromogen (Vector).

### Frozen section immunohistochemistry with prefixation

This method was used to prepare material for immunohistochemistry of markers tolerant to paraformaldehyde fixation as ED-1 (anti-CD68, Serotec), DC11 or MN423 (Axon Neuroscience) and for stereological analysis. Rats were deeply anaesthetised with ketamine-xylasine and perfused intracardially using a peristaltic pump: for 1 min with phosphate-buffered saline (PBS) and for 7 min with 4% paraformaldehyde in PBS, pH 7.2 (4% PFA). The brain was post-fixed overnight in 4% PFA, cryoprotected with 15% and 25% sucrose solutions (subsequently overnight), frozen in 2-methylbutane (30 seconds at -42°C) and transferred to dry ice. Sagittal brainstem sections (40 μm thick) were cut on a Leica CM1850 cryomicrotome.

For stereological analysis, every 13th section was included in the series (section sampling fraction = 1/13, total 17-19 sections per animal). Tissue sections were incubated with primary antibodies AT8 (Pierce Endogen), OX-6 (anti-MHCII, Serotec), anti-Iba1 (WAKO, USA), anti-NeuN antibody (Chemicon, USA) or anti- GFAP (Dako, Belgium) overnight at 4°C. Sections were immunostained using the standard avidin-biotin-peroxidase method (Vectastain ABC kit) with VIP as chromogen and 5 minute counterstaining of cell nuclei by methyl green (Vector).

### Stereological quantification

For stereological quantification, male rats of transgenic lines SHR TG (N = 5) and WKY TG (N = 5), in the terminal stage of transgenic phenotype development, were used along with their non-transgenic littermates as age-matched controls (SHR crl, N = 4 and WKY crl, N = 6). The numbers of Iba1-positive microglia, MHCII-positive microglia, neurofibrillary tangles, neurons and astrocytes were quantified by stereology in brainstem (pons and medulla oblongata). This region of interest was chosen because it is highly affected by the neurofibrillary pathology in the brain of these tau transgenic rats.

The study was accomplished using an optical fractionator approach on a modified light microscope (Olympus BX51) equipped with a computer-based stereological system (StereoInvestigator; MicroBrightField, Williston, VT, USA). The region of interest was selected at low magnification (objective 4 × UPlanFI), and counting was performed at high magnification using an objective with a high numerical aperture (60 × oil immersion objective, Olympus, UPlanFI, NA = 1.25) and oil condenser (Olympus, UAAC, PlanApo, NA = 1.40). Parameters of the stereological analysis (disector base, sampling grid) were chosen according to density of the particular structure (e.g. NFT, microglia) in the tissue - sparse NFTs required a denser grid than did frequent microglia to ensure precise analysis with appropriately low coefficient of error. Detailed parameters of the stereological analysis are described in Table 1.

**Table 1:** Parameters of the stereological analysis

			dissector		sampling						
			base	height	Grid	UGZ	BA	t			
Structure quantified (antibody)	OBJ	ssf	(μm × μm)	(μm)	(μm × μm)	(μm)	(μm)	(μm)	∑OD	∑Q-	CE
Neurofibrillar tangles (AT8)	60 ×	1/13	100 × 100	15	370 × 370	1.5	40	21.85	3296.70	394.80	0.060
Microglia (Iba1)	60 ×	1/13	70 × 70	15	860 × 860	1.5	40	24.41	767.20	1115.40	0.030
Macrophage-like structures (Iba1)	60 ×	1/13	70 × 70	15	860 × 860	1.5	40	24.41	767.20	96.00	0.260
MHCII-positive microglia - frequent (MHCII)	60 ×	1/13	100 × 100	15	500 × 500	1.5	40	25.60	2645.40	1861.40	0.041
MHCII-positive microglia - sparse (MHCII)	60 ×	1/13	100 × 100	15	370 × 370	1.5	40	26.54	4029.20	252.20	0.085
Astrocytes (GFAP)	60 ×	1/13	70 × 70	15	860 × 860	1.5	40	24.66	680.19	1696.05	0.024
Neurons (NeuN)	60 ×	1/13	70 × 70	15	650 × 650	1.5	40	20.70	1243.76	1655.81	0.047

Abbreviations: OBJ - objective; ssf - section sampling fraction; UGZ - upper guard zone; BA - block advance (section thickness when cut); t - average number-weighted section thickness after immunohistochemical processing; ∑OD - average sum of optical dissector used; ∑Q- - average number of markers counted; CE - coefficient of error (Schmitz-Hof).

### Statistical analysis

All data sets are expressed as mean ± standard error of the mean (SEM). Results were examined using an unpaired Student's t-test (with Welch's correction in case of uneven variances) and two-way ANOVA using Prism GraphPad Software Version 4.03 (Graph Pad Software, Inc., USA). Differences were considered to be statistically significant if p < 0.05.

## Results

### Back-crossed WKY TG rats remain normotensive and maintain WKY habitus

**Figure 1:**
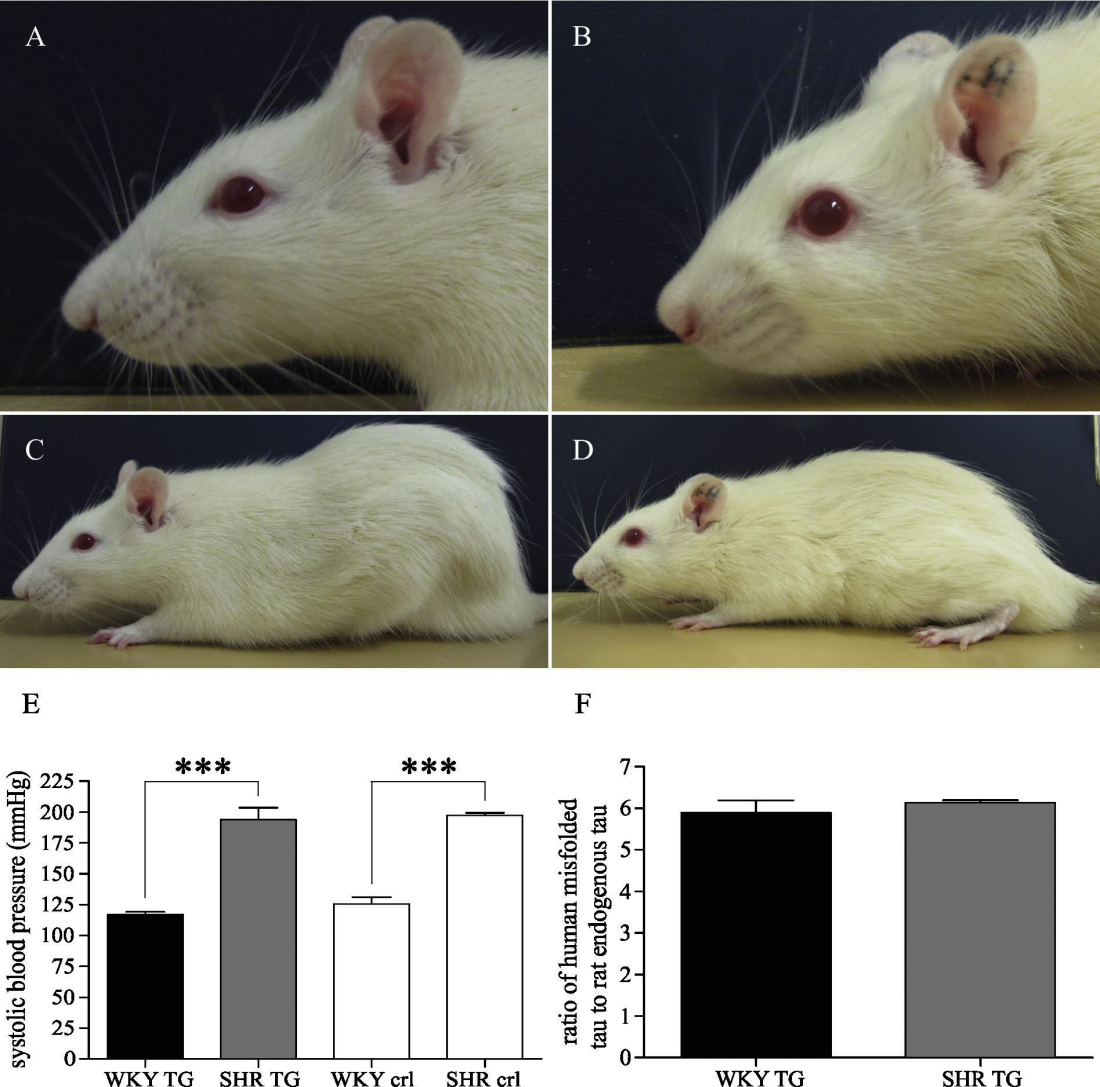
**Back-crossed WKY TG rats show the basic features of the WKY strain**. Rats of the WKY TG line in the F5 generation demonstrated morphological features of the WKY strain, including elongated skull shape (A) and increased body size (C), compared to SHR rats (B, D). Systolic blood pressure measurements show chronic hypertension in SHR controls and SHR TG rats, and confirm normal blood pressure in WKY controls as well as in the back-crossed WKY TG line (E). The influence of genetic background factor on systolic blood pressure is highly significant (two-way ANOVA, F = 166.85, *** p < 0.0001), but transgenic factor (F = 1.14, p = 0.30) or their interaction (F = 0.2, p = 0.66) does not influence the blood pressure of the rats. Transgene expression (measured as ratio of human misfolded to rat endogenous tau) is almost identical in the brains of SHR TG and WKY TG rats (F).

The SHR TG line was back-crossed to the WKY genetic background. WKY TG rats in the F5 generation showed typical WKY habitus (Figure 1A, C) characterized by elongated skull shape and increased body size and body weight compared to SHR rats (Figure 1B, D). At 24 weeks, the mean body weight was 425 g ± 18 in WKY TG rats, 501 g ± 14 in WKY controls, 295 g ± 8 in SHR TG rats and 337 g ± 7 in SHR non-transgenic littermates. While SHR TG and SHR control rats suffered chronic hypertension, WKY TG remained normotensive as did WKY control rats (Figure 1E). Transgene expression, measured as human misfolded tau to rat endogenous tau ratio, was equivalent in both transgenic lines (Figure 1F) (Student's t-test with Welch's correction, t = 0.81, df = 3, p = 0.48).

### Neurofibrillary pathology displays similar qualitative but different quantitative profiles in two transgenic rat models of tauopathy (SHR and WKY)

**Figure 2:**
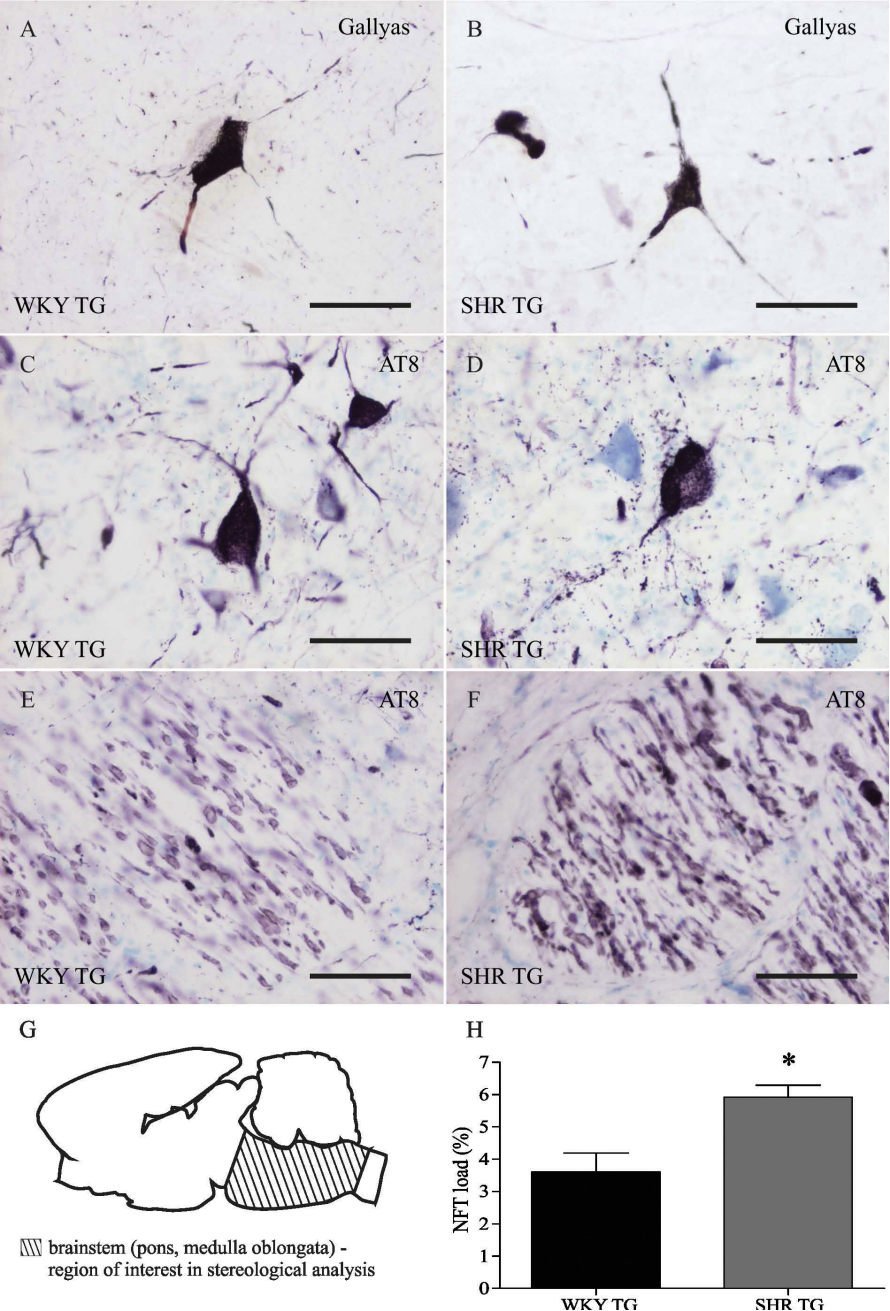
**Qualitative and quantitative profile of neurofibrillary pathology in two transgenic rat models of human tauopathy**. NFTs show similar morphological and immunohistochemical features in both transgenic lines - WKY TG (A, C) and SHR TG (B, D). Neurofibrillary tangles were stained by Gallyas silver staining (A, B, reticular nuclei) and immunolabeled with antibody AT8 recognizing tau phosphorylated at Ser202 and Thr205 (C, D, reticular nuclei). Phosphorylated tau is also distributed in neuronal processes, resulting in axonopathy visible in the white matter (E, F). The number of NFTs was stereologically quantified in brainstem (pons and medulla oblongata) as a region of interest (G). Stereological analysis revealed significantly lower number of NFTs in WKY TG rats compared to SHR TG rats (H, Student's t-test, * p < 0.05). Pre-fixed frozen sections. Scale bars: 50 μm.

Argyrophilic neurofibrillary tangles (NFTs) and axonal degeneration were prominent in the brainstem and spinal cord of transgenic SHR and WKY rats. Neurofibrillary pathology was detected by Gallyas silver staining (Figure 2A, B) and immunolabeled with monoclonal antibody AT8 that recognizes tau protein phosphorylated at Ser202 and Thr205 (Figure 2C-F). Immunostaining with antibodies that label conformationally modified tau (DC11, MN423) did not reveal significant differences between transgenic rat lines (data not shown). Despite similar transgene expression levels in the brainstems of both lines, stereological analysis of terminal-staged transgenic rats revealed a significantly lower number of AT8-positive NFTs in the brainstem of WKY TG line than in SHR TG (Student's t-test, t = 2.67, df = 8, p < 0.05) (Figure 2G, H). The difference was approximately 1,6-fold.

### Genetic background determines neuroinflammatory pattern in transgenic rats

**Figure 3:**
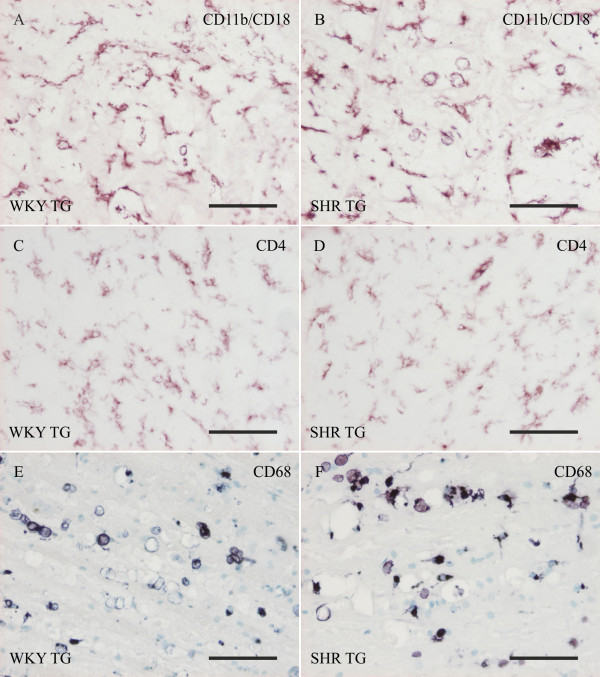
**Qualitative profile of activated microglia in two transgenic rat models of human tauopathy**. Microglia in brainstem of WKY TG (A, C, E) as well as SHR TG (B, D, F) rats show increased expression of activation markers. Large number of microglia strongly immunoreactive for CD11b/CD18 (complement 3 receptor) (A, B) demonstrate the involvement of complement system in the tau-induced neuroinflammation. CD4 co-receptor is usually present on T-cells interacting with MHCII-positive antigen presenting cells; in tau-induced neuroinflammation it is increased mainly on microglia/macrophages (C, D). Round-shaped cell parts positive for CD68 (lysosomal membrane glycoprotein) indicate putative phagocytosis (E, F). Post-fixed (A, B, C, D) and pre-fixed (E, F) frozen sections. Scale bars: 50 μm.

**Figure 4:**
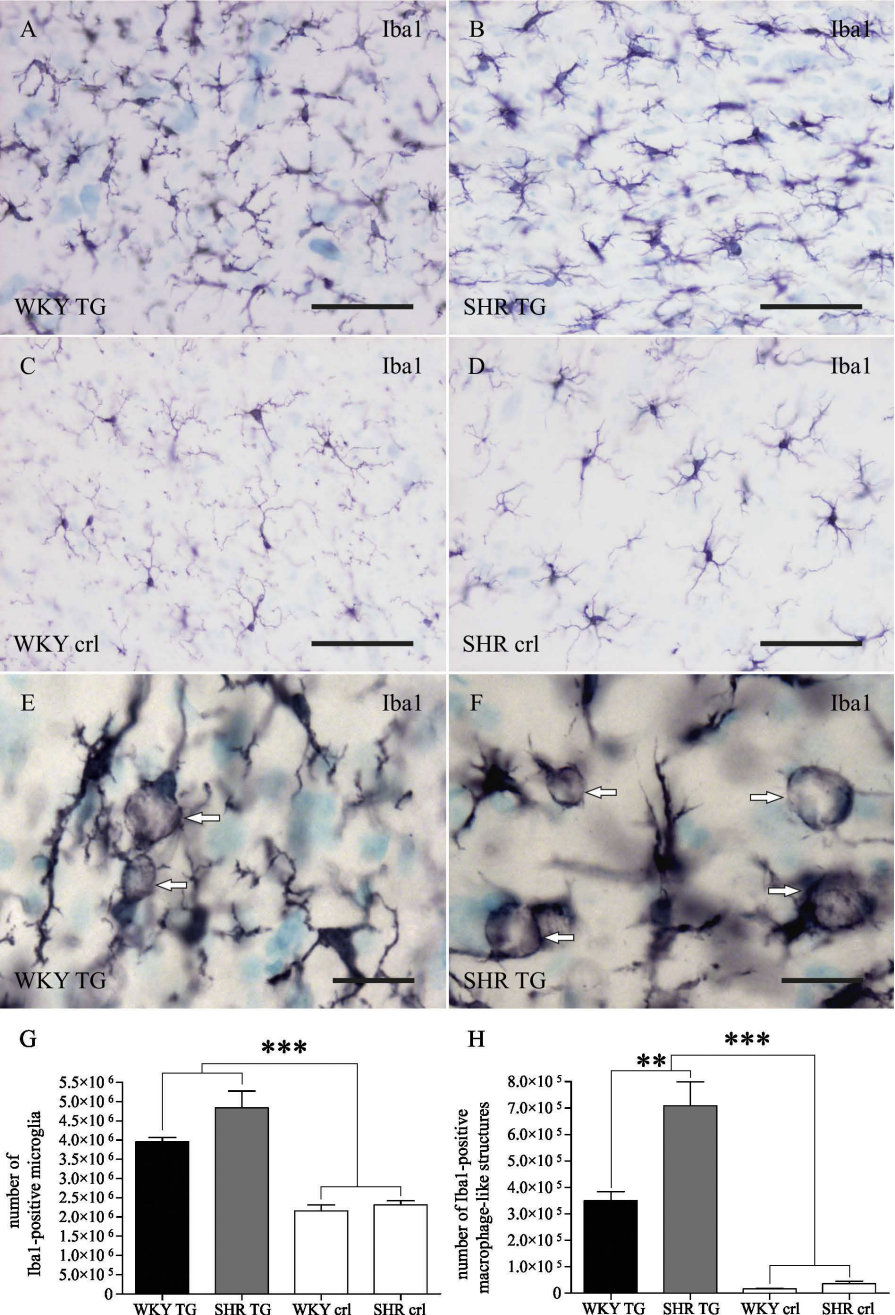
**Qualitative and quantitative profile of activated microglia in two transgenic rat models of human tauopathy**. Prominent microgliosis was observed by immunohistochemical staining with Iba1 antibody in both transgenic lines - WKY TG (A) and SHR TG (B) - compared to non-transgenic WKY (C) and SHR (D) age-matched controls. Stereological quantification revealed that the numbers of Iba1-positive microglia/macrophages in brainstem of both transgenic lines doubled in comparison with non-transgenic controls (G, two-way ANOVA, transgenic factor, *** p < 0.0001). There is also a tendency to a higher number of Iba-1 positive microglia in SHR TG rats compared to WKY TG. The morphology of microglia in brainstem of transgenic rats indicates a high level of activation including presumptive phagocytosis (E, F - arrows). In SHR TG rats, there are significantly more microglia showing phagocyte-like morphology than in WKY TG (H, two-way ANOVA, transgenic factor, *** p < 0.0001, genetic background factor, ** p = 0.001). Pre-fixed frozen sections. Scale bars: 50 μm for A-D, 20 μm for E, F.

In brain areas harbouring neurofibrillary pathology and axonopathy, extensive inflammation mediated by reactive microglia was observed. In both transgenic lines, immunohistochemical staining showed activated morphology of microglia (thick shortened processes, macrophage-like morphology, forming of cell clusters) and increased microglial expression of activation markers: CD4, CD11b/CD18 and CD68 (Figure 3). Stereological quantification of cells stained by the general microglial marker Iba1 was performed in both transgenic lines and age-matched controls (Figure 4). The terminal stage of the transgenic phenotype was characterized by a two-fold increase in Iba1-positive microglia compared to control WKY or SHR rats (Figure 4G). Two way ANOVA showed that the transgenic factor creates approximately 74.5% of variability in microglial counts (F = 73.08, p < 0.0001), while the factor of genetic background demonstrates only a non-significant trend towards increased number of Iba1-positive microglia in SHR TG rats and control rats compared to WKY TG and WKY controls (F = 4.198, p = 0.057). The results were not significantly influenced by the interaction of examined factors (F = 2.06, p = 0.17). In the stereological quantification of the Iba1-positive cells, phagocytic morphology (Figure 4E, F - highlighted by arrows) was likewise considered. Macrophage-like structures were frequently observed in transgenic brainstems, but occasionally seen in control rats. The average ratio of phagocytic morphology observed among Iba1-positive cells was 8.9% in WKY TG rats, 14.5% in SHR TG, 0.8% in WKY controls and 1.5% in SHR control rats. Phagocytic morphology followed a similar but more pronounced pattern as stereological quantification of the number of the Iba1-positive microglia, and showed significant differences between WKY TG and SHR TG rats (Figure 4H). In two-way ANOVA analysis, the transgenic factor accounted for 69% of variability in the number of macrophage-like structures (F = 106.7, p < 0.0001). However, the factor of genetic background was responsible for 9.85% of the variability (F = 15.13, p = 0.001). Interaction (F = 12.17, p = 0.003) also influenced the result significantly, indicating that genetic background did not have the same effect on the number of macrophage-like structures in transgenic and control rats.

### WKY TG rats express strikingly more MHCII-positive microglia than SHR TG

**Figure 5:**
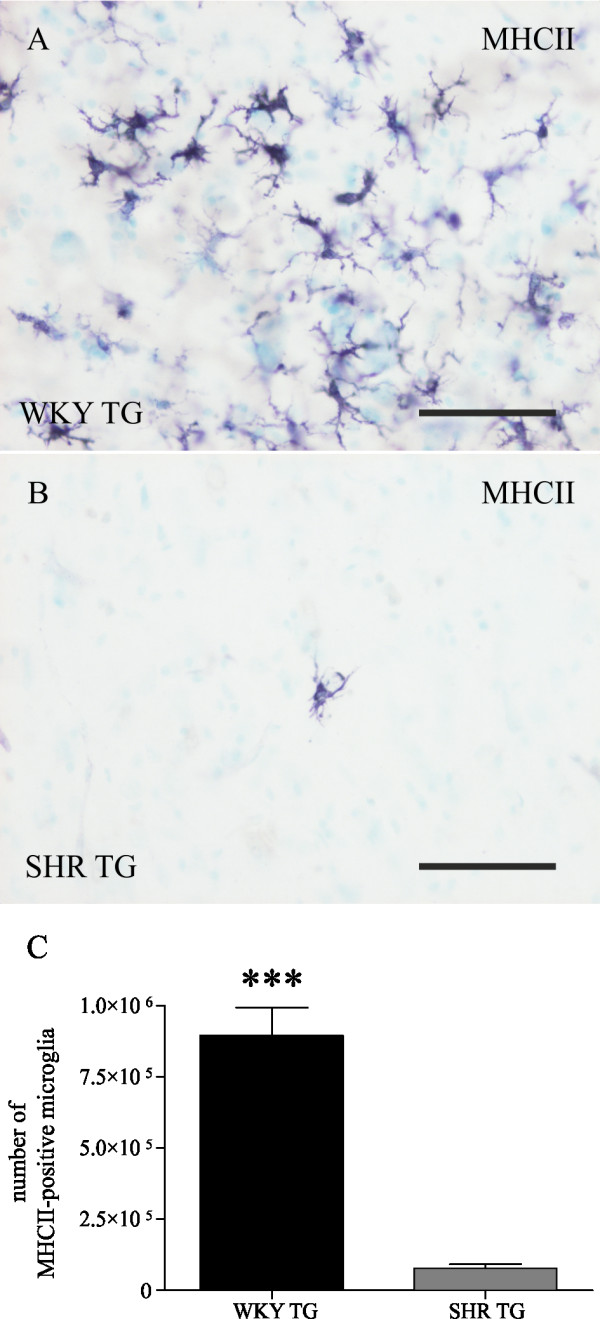
**Expression of MHC class II molecules is different in brains of transgenic rat models**. In brainstem of WKY TG rats (A), activation of microglia is accompanied by widespread MHCII expression, while in SHR TG (B), only sparse MHCII staining was recorded. Stereological quantification shows highly significant differences between the transgenic lines. In WKY TG rats, there are 10 times more microglia that express MHCII than are present in SHR TG rats (C, Studen't t-test, *** p < 0.001). Pre-fixed frozen sections. Scale bars: 50 μm.

Brainstems of transgenic rats were stained for MHC class II antigens - RT1B (Figure 5.). Quantification of non-transgenic rats was not undertaken due to lack of MHCII-positive cells in several control brainstems. A striking difference in the number of MHCII-immunoreactive microglia (Student's t-test, t = 8.2, df = 8, p < 0.0001) was detected between the genetic backgrounds, showing different types of microglial activation. In WKY TG, 23.2% of total (Iba1-positive) brainstem microglia expressed MHCII, while in SHR TG rats, only 1.6% of total microglia were MHCII-positive, respectively.

### The increase in astrocyte numbers is the same in both transgenic rat lines regardless of the genetic background

**Figure 6:**
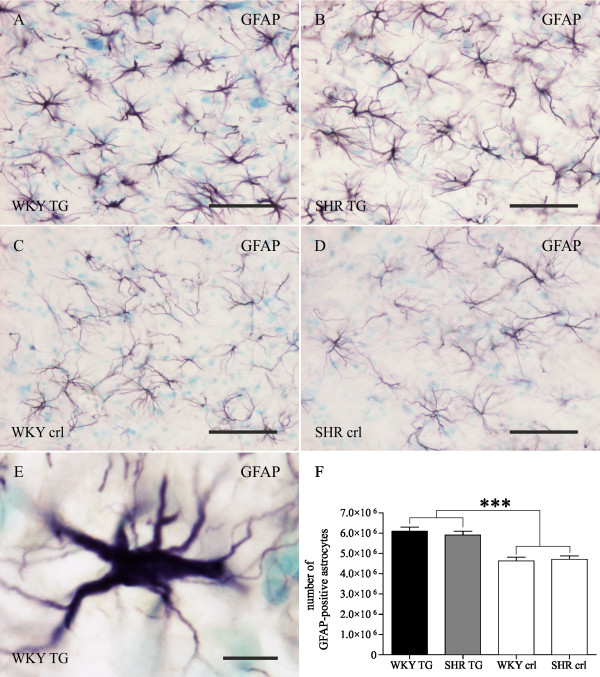
**Qualitative and quantitative profile of activated astroglia in two transgenic rat models of human tauopathy**. There is an approximately 25% increase in GFAP-positive astrocytes in brainstem in both transgenic lines (A - WKY TG, B - SHR TG) compared to age-matched controls (C - WKY crl, D - SHR crl). Morphological signs of astrocytic activation and hypertrophy (E) are observed in the brainstems of both transgenic rat lines. Stereological quantification (F) reveals no significant differences between WKY TG and SHR TG rats in numbers of GFAP-positive astrocytes. However, the difference in numbers of GFAP-positive astrocytes between transgenic and control rats is highly significant (two-way ANOVA, transgenic factor *** p < 0.0001). Pre-fixed frozen sections. Scale bars: 50 μm for A-D, 20 μm for E.

Besides microglial activation, the reaction of astrocytes was observed and quantified (Figure 6). Independently from the genetic background, an elevated number of GFAP-positive astrocytes were counted in both lines of transgenic rats (SHR TG and WKY TG) in comparison with non-transgenic age-matched controls. Two-way ANOVA revealed a significant role of the transgenic factor impacting 71.53% of variability (F = 71.53, p < 0.0001), while genetic background (F = 0.1, p = 0.80) or interaction (F = 0.71, p = 0.51) did not influence the number of GFAP-positive astrocytes.

## Discussion

Neurofibrillary degeneration [25,26] and neuroinflammation [27-29] are prominent features of Alzheimer's disease (AD) and related neurodegenerative disorders. While neurodegeneration induced by misfolded tau correlates with disease progression [30], inflammation is considered to be an important factor in the variability in resistance or susceptibility to AD [5,31]. Polymorphisms of genes connected to inflammatory pathways are often among the AD risk factor candidates [32-34]. A recent transcriptomic study of centenarians has revealed that immune homeostasis determines cognitive health of the population aged over 90 years. Genes related to immune response pathways, particularly antigen presentation, IL4, natural killer cell signalling, complement pathway and acute phase signalling are upregulated in cognitively intact oldest-old (compared to younger adults), while downregulated in cognitively impaired elderly. Hence, genes related to immunity and regulation of their expression significantly influence the pathogenesis of neurodegeneration [35].

In this study, we have shown that misfolded tau protein induces neurofibrillary degeneration regardless of genetic background. However, the immune response driven by neurodegeneration was significantly determined by genetic modifiers.

Two transgenic lines raised on different genetic backgrounds (SHR, Wistar-Kyoto) were utilized expressing the same amount of human truncated tau. Despite the fact that the tau expression profile was similar in both transgenic lines, the final load of neurofibrillary tangles was significantly different. Stereological quantification revealed lower final NFT load in the brainstem of transgenic WKY rats compared to transgenic SHR counterparts. Previously, we showed that the final NFT load in the terminal stage was independent of transgene expression levels [36]. Altogether, these findings suggest that the final NFT load is independent of expression levels of misfolded tau protein, but remains strongly dependent on the interaction of the transgene and genetic environment.

Neuroinflammation represents one of the key differences between SHR and Wistar strains. Spontaneously hypertensive rats (SHR) exhibit chronic neuroinflammation [37,38]. RT-PCR analysis demonstrates significantly increased expression of TNFα, ICAM-1, iNOS and the inflammation-related nuclear transcription factor PPARγ in brain in SHR compared to that in WKY rats [39]. In addition, the brain endothelium of SHR is more sensitive to cytokines; under inflammatory conditions it is more adhesive to blood monocytes in comparison with WKY endothelium [40,41]. On the other hand, the chronic inflammation in SHR rats probably causes an altered reaction to acute immune challenges. These rats show an attenuated response to endotoxin shock or polymicrobial infection, and survive these challenges better than do WKY rats [42,43]. In order to identify the impact of neuroinflammation on tau neurodegenerative cascade, we analyzed the pattern of microglial activation in both transgenic lines (SHR TG and WKY TG). We discovered that both transgenic rat models demonstrated the same distribution and immunophenotypic profile of activated microglia. Despite profound similarities between both transgenic lines, several features of neuroinflammation revealed striking differences that were driven by their genetic background (Table 2.). Transgenic SHR rats displayed increased number of microglia and significantly more Iba1-positive cells with phagocytic morphology than WKY transgenic rats. In human AD brain, Aβ-phagocytosing cells operate efficiently in healthy brain, but are inefficient in AD patients. These Aβ-phagocytosing cells appear to be mainly blood-borne macrophages [44,45]. Similarly, we have shown that at least some of the cells with phagocytic morphology in our model comprise macrophages infiltrating from the blood [20], suggesting that macrophages may play important role in the immune response in the transgenic rats expressing truncated tau.

**Table 2:** Pattern of the tissue response

	AT8-positive	Iba1-positive	Iba1-positive	MHCII-positive	GFAP-positive
	NFTs	microglia	phagocytes	microglia	astrocytes
WKY TG	++	++(+)	++	+++	++
SHR TG	+++	+++	+++	+	++

Pattern of the tissue response on the tau-induced neurodegeneration in the terminal stage of transgenic phenotype, revealed by stereological quantification.

The most profound difference was found in the number of MHC class II positive microglial cells. In WKY transgenic rats, 23.2% of Iba1-positive microglia were MHCII-positive, while in transgenic SHR rats, only 1.6% of microglia expressed MHCII. Microglial expression of MHCII is considered to be a response to phagocytosis of tissue debris, involved in activating antigen cleavage machinery [46]. However, in our model, SHR transgenic rats showed a higher number of cells with phagocytic morphology but only a sparse MHCII signal. A similar discrepancy between MHCII expression and markers of phagocytosis has been described for a model of spinal cord injury. These authors demonstrated that Lewis rats exhibit more antigens related to complement and phagocytosis, while Sprague Dawley rats express more MHCII antigens [47]. We suppose that the tendency to increased microglial numbers and significantly more phagocytic morphology in SHR microglia may be a compensatory reaction for the inability to amplify MHCII expression.

The functional impact of microglial MHCII expression on neurodegeneration is not yet fully understood. Models of CNS injury reveal that extensive MHCII expression correlates with tissue damage. In a ventral root avulsion model [48], MHCII expression accompanies increased motoneuron loss. Furthermore, rat strains with higher MHCII expression are also more susceptible to experimental autoimmune encephalomyelitis. Similarly, Kigerl et al. [49] reported impaired recovery and more extensive lesion volume in mouse strain with the highest levels of MHCII in a model of spinal cord injury. In our study, WKY TG rats, displaying increased numbers of MHCII positive microglia, showed higher vulnerability to tau-induced neurodegeneration compared to SHR TG rats: lower burdens of neurofibrillary pathology sufficed for WKY TG rats to reach the terminal stage of transgenic phenotype. These results suggest that microglia expressing MHCII can considerably modify the progression of neurodegeneration. This hypothesis should be validated by further experiments including assessment of functional behavioural changes and morality.

Besides microglial activation, we also observed increased numbers of activated hypertrophic astrocytes in both transgenic rat lines. Astrogliosis - astrocytic activation associated with inflammatory signaling and decrease in trophic support of neurons - is a common feature of neurodegenerative conditions, including human AD and transgenic models with amyloid or tau pathology [50-58]. Surprisingly, in our experimental conditions, the increase in number of GFAP-positive astrocytes was not influenced by genetic background. However, we cannot exclude that the genetic environment could modify the functional properties of activated astrocytes.

## Conclusions

In this report, we have shown for the first time that genetic background has a strong influence on the load of neurofibrillary tangles and the inflammatory pattern of activated microglia. These findings suggest that brain immune response is a potent disease modifier of neurofibrillary degeneration. We therefore conclude that targeted immunomodulation rather than anti-inflammatory strategies may represent a prospective therapeutic avenue for Alzheimer's disease.

## Competing interests

The authors declare that they have no competing interests.

## Authors' contributions

ZS contributed by designing the stereological study, blood pressure measurements, tissue preparation, histological and immunohistochemical staining, stereological quantification of Iba1, MHCII and GFAP-stained slides; statistical analysis and writing of the manuscript. NZ conceptualized and implemented the idea of back-crossing the SHR TG line to the WKY background, and helped with data interpretation for, critical reading of, and key suggestions for the manuscript. PN carried out the stereological quantification of AT8 and NeuN-stained slides. BK accomplished proteomical part of the study. OB set up the stereological system and prepared tissue samples for immunohistochemical staining. MN was the leading, coordinating, and decisive mind of the project, and shaped the final form of the manuscript. All authors read and approved the final manuscript.
